# Recrudescent *Plasmodium berghei* from Pregnant Mice Displays Enhanced Binding to the Placenta and Induces Protection in Multigravida

**DOI:** 10.1371/journal.pone.0005630

**Published:** 2009-05-20

**Authors:** Claudio R. F. Marinho, Rita Neres, Sabrina Epiphanio, Lígia A. Gonçalves, Manuela Beirão Catarino, Carlos Penha-Gonçalves

**Affiliations:** 1 Instituto Gulbenkian de Ciência, Oeiras, Portugal; 2 Unidade de Malária, Instituto de Medicina Molecular, Universidade de Lisboa, Lisboa, Portugal; 3 Faculdade de farmácia, Universidade de Lisboa, Lisboa, Portugal; 4 Departamento de Ciências Biológicas, Universidade Federal de São Paulo, Diadema, Brazil; Université Pierre et Marie Curie, France

## Abstract

Pregnancy-associated malaria (PAM) is associated with placenta pathology and poor pregnancy outcome but the mechanisms that control the malaria parasite expansion in pregnancy are still poorly understood and not amenable for study in human subjects. Here, we used a set of new tools to re-visit an experimental mouse model of pregnancy-induced malaria recrudescence, BALB/c with chronic *Plasmodium berghei* infection. During pregnancy 60% of the pre-exposed primiparous females showed pregnancy-induced malaria recrudescence and we demonstrated that the recrudescent *P. berghei* show an unexpected enhancement of the adherence to placenta tissue sections with a marked specificity for CSA. Furthermore, we showed that the intensity of parasitemia in primigravida was quantitatively correlated with the degree of thickening of the placental tissue and up-regulation of inflammation-related genes such as *IL10*. We also confirmed that the incidence of pregnancy-induced recrudescence, the intensity of the parasitemia peak and the impact on the pregnancy outcome decreased gradually from the first to the third pregnancy. Interestingly, placenta pathology and fetal impairment were also observed at low frequency among non-recrudescent females. Together, the data raise the hypothesis that recrudescent *P. berghei* displays selected specificity for the placenta tissue enabling on one hand, the triggering of the pathological process underlying PAM and on the other hand, the induction of PAM protection mechanisms that are revealed in subsequent pregnancies. Thus, by exploiting *P. berghei* pregnancy-induced recrudescence, this experimental system offers a mouse model to study the susceptibility to PAM and the mechanisms of disease protection in multigravida.

## Introduction

It is estimated that more than 50 million pregnancies occur in malaria endemic areas per year, and approximately half of these occur in sub-Saharan Africa, where *Plasmodium falciparum* transmission is most intense [1]. In these regions adults tend to become protected from severe forms of the disease and from high-density parasitemia. Such protection appears to be partially lost during pregnancy when *P. falciparum* infection frequently courses with maternal and fetal morbidity leading to over 100.000 infant deaths in Africa every year [2]. Besides experiencing the common malaria symptoms, pregnant women suffer higher abortion rates and their offspring manifest intrauterine growth retardation in combination with low birth weight, which is a known risk factor for neonatal mortality [3], [4].

In pregnancy-associated malaria (PAM) the occurrence of stillbirths, premature and low-birth weight deliveries is attributable in part to maternal malaria-induced anemia [5], but the risk of low birth weight approximately doubles if women have placental malaria [6]. The infection of the placenta is characterized by accumulation of infected red blood cells (iRBC) in the intervillous spaces that, together with the concurrent placental pathology, could lead to fetal development impairment [7]. It has been proposed that the combined effect of parasite sequestration, massive intervillous inflammatory infiltration, thickening of the trophoblastic basement membrane and cytotrophoblast proliferation may disturb the transport of oxygen and nutrients across the placenta, impairing the development of the fetus [7].

It is widely accepted that a key component of the severe forms of *P. falciparum* malaria is the ability of the iRBC to adhere and be sequestered in various tissues. This is due to the interaction between parasite proteins expressed on the iRBC membrane and a variety of host receptors. In PAM, candidate host receptors for placental iRBC sequestration include a glycosaminoglycan, the low-sulphated chondroitin sulphate A (CSA) [8], which is spread throughout the intervillous spaces [9], [10]. Furthermore, it was shown that the gene *var2csa* is highly expressed in placental parasites [11] and confers enhanced CSA adhesion and specific antigenic properties to the iRBC [12]. These distinct antigens expressed by placental *P. falciparum* are considered major targets for acquired protective immunity to PAM [13], [14].

Epidemiological data strongly support the hypothesis that acquired immunity to placental *P. falciparum* is an effective protection mechanism in PAM [15]. Thus, in areas of high malaria transmission, it has been repeatedly observed that the risk of PAM decreases in multigravida. The severe clinical manifestations are mainly restricted to the first and second pregnancies [16], when the levels of specific antibodies against placental parasite antigens are not high enough to confer protection [17], [18]. Interestingly, in areas of infrequent malaria exposure, where women have little or no malaria immunity, the disease results in severe outcomes both for the mother and the baby, irrespective of parity [9], reinforcing the notion that the severity of clinical manifestations during pregnancy depends on pre-existing immunity of the mother [19]. Thus, malaria exposure through consecutive pregnancies is required for placental malaria immunity to develop and be maintained [15].

Nevertheless, in high transmission areas women that are exposed to malaria before pregnancy may show increased susceptibility to malaria during pregnancy but the parasite and host mechanisms that lead to parasite recrudescence are difficult to address in human populations. In addition, malaria can be asymptomatic during pregnancy and have serious consequences for the developing fetus [9], [20] suggesting that complex pathogenesis mechanisms are linking parasite recrudescence, placenta pathology and pregnancy outcome. Moreover, the biological basis of the protection acquired by multigravida needs to be investigated in experimental systems that warrant absence of re-infection.

In the 1980 s, van Zon and Eling provided seminal descriptions of pregnancy-induced malaria recrudescence in mice [20], [21] and noted higher vulnerability to malaria in pregnant female mice. Recently, we have shown that the main pathological features of severe PAM are reproduced in a mouse model [22] and Megnekou et al. [23] evidenced that pregnant mice accumulated specific antibodies against recrudescent parasite. These reports point to the value of mouse models as a research tool in pregnancy malaria. Here we extended our studies on experimental PAM and investigated whether pregnancy-induced *P. berghei* ANKA recrudescence correlated with placenta pathology, decreased with parity and was associated with enhanced binding of the recrudescent parasite to placental tissue.

## Materials and Methods

### Animals, parasites and infection

BALB/c mice were bred and maintained in conventional housing and fed with regular diet. All procedures were in accordance with national regulations on animal experimentation and welfare, authorized by the Instituto Gulbenkian de Ciência animal welfare committee. Infection experiments were performed in adult females, between 10–15 weeks of age. P. berghei ANKA constitutively expressing green fluorescent protein (*P. berghei* ANKA-GFP) (259Cl2 clone) [24], [25]. The mice were infected intra-peritoneally (IP) with 10^6^ iRBC obtained from frozen stocks and treated IP with 0.7 mg chloroquine for 3 days, starting at day 7 post-infection [26] and parasitemia was recorded every other day using flow cytometry analysis as described elsewhere [27]. Five to ten percent of the female mice exposed to this treatment succumbed but the remaining recovered from the infection after chloroquine therapy and were used in pregnancy-induced recrudescence experiments.

### Parasite recrudescence and offspring monitoring

Forty days post-infection a group of treated females were put to mate and the remaining were used as non-pregnant controls. Detection of the vaginal plug and measurement of body weight were jointly used to time gestation, as previously described [28]. The day of vaginal plug detection was considered as gestational day one (G1). Pregnancy was confirmed between G10 and G13 when the animals had an average increase of 3–4 g in body weight. Thus, weight gain was taken as sign of pregnancy and sudden weight loss as an indicator of pregnancy injury or interruption. Some of the pregnant females were subjected to caesarian section at G19 for placenta pathology studies, while the others were allowed to deliver and to follow to subsequent pregnancies. At delivery, the weight and the number of live newborns were registered. Newborns weight and development was followed up to day 30 after birth. Non-infected pregnant females were used as controls.

### Placentas collection and histopathological analysis

Females were killed by CO_2_ narcosis at G19. Placentas from infected and non-infected pregnant females were treated in a similar way. Placentas were separated in two halves, one half was frozen for RNA extraction and the other was fixed in 10% formalin for further processing. Paraffin-embedded non-consecutive placenta sections were stained with hematoxylin-eosin (HE) and examined in light microscopy (Leica DM LB2, Leica Microsystems). For histological and morphometric analysis, placental sections were blindly examined as previously described [22].

### Gene-Specific Expression by qRT-PCR

Total RNA, from individual placentas and viable newborns, was obtained using an RNeasy Mini Kit (Qiagen), following the manufacturer's protocol for animal tissues. One microgram of total RNA was converted to cDNA (Transcriptor First Strand cDNA Synthesis Kit, Roche) using random hexamer primers. MCP-1 (*Ccl2*) and MIP-1α (*Ccl3*) expression was quantified using TaqMan Gene Expression Assays from ABI (Mm00441242_m1 and Mm00441258_m1, respectively) with TaqMan Universal PCR master mix. T lymphocytes (*Cd3e*), natural killer cells (*Klrd1*), macrophages (*Mgl2*), neutrophils (*Ncf2*), cytokines and hemoxygenase-1 (*Hmox-1*) expression was amplified using primer sequences previously described [29]. Endothelin-1 (*Edn1*) and β-actin (*Actb*) specific primer sequences were, *Edn1* -5′-ACG CAC AAC CGA GCA CAT TGA CTA C-3′and 5′ TCC TGC CCG TCT GAA CAA GAA ACT G-3′ and *Actb* - 5′ AGC CAT GTA CGT AGC CAT CC-3′ and 5′-CTC TCA GCT GTG GTG GTG AA-3′. These qRT-PCR reactions used Applied Biosystems Power SYBR Green PCR Master Mix. The gene expression quantification reactions were performed according to the manufacturers' instructions on an ABI Prism 7900HT system. Relative quantification of specific mRNA was normalized for a mouse housekeeping gene mRNA. To select an appropriate internal control, we studied the expression of the following housekeeping genes: ACTB, GAPDH, TATA box binding protein (TBP), Succcinate dehydrogenase complex, subunit A (SDHA) and Tyrosine 3-monooxygenase/tryptophan 5-monooxygenase activation protein, zeta polypeptide (YWHAZ). The last three genes have been previously validated in human placental malaria [30]. Due to uneven gene expression, when comparing non-infected and infected placentas, the housekeeping genes TBP, SDHA and YWHAZ were unsuitable for internal controls. Conversely, ACTB and GAPDH expression was maintained under infection conditions.

### Synchronization of parasitized erythrocytes

Infected red blood cells were obtained from infected non-pregnant mice, and from pregnant females with recrudescence, having 10–20% parasitemia. In order to obtain mature blood stage parasite forms (trophozoites/ schizonts), *P. berghei* ANKA-GFP infected erythrocytes were synchronized as described elsewhere [31]. Usually, the mature forms enrichment yields over 90% of infected cells. The enriched infected erythrocytes preparations were suspended in PBS at a concentration of 10^8^ iRBC/ml.

### iRBC binding assays in placental sections

Placentas from uninfected BALB/c females, obtained at G19, were treated using a previously described protocol [10]. Briefly, the placentas were fixed in 2% formalin and 0.5% glutaraldehyde for 10 minutes, heated in a microwave oven before being paraffin-embedded, cut into sections of 5 µm onto glass slides and then deparafinized and rehydrated. This fixation protocol aims to preserve the binding capacity of glycosaminoglycans in the placenta intervillous spaces. For placenta-receptor cleavage experiments, placental sections were incubated with 0.5 U/ml chondroitinase ABC (from *Proteus vulgaris*, Sigma) or heparinase II (from *Flavobacterium heparinum*, Sigma) as a negative control, or with PBS, for 2 periods of 2 hours at 37°C. Fifty microliters of synchronized iRBC suspension were overlaid onto each tissue section for 60 minutes at 37°C in a humid chamber. For iRBC-ligand blocking experiments, synchronized iRBC were preincubated, for 30 minutes at 37°C, with CSA from bovine trachea (Sigma) or colominic acid sodium salt (CA) from *E. coli* (Sigma) as negative control, using the concentration of 1 mg/ml for each. This concentration was chosen to achieve maximum binding inhibition, while lower concentrations such as 100 mg/ml led to about 50% inhibition of specific binding (data not shown). The slides were mounted with Mowiol and examined under fluorescence microscopy at 40× magnification. The number of fluorescent green fluorescent iRBC adhering placenta sections in each experimental condition was determined in a blind fashion and counting for each section 50 microscopic fields.

### Statistical analysis

Statistical differences between groups of mice were evaluated by the Student-t or Mann-Whitney tests. Chi-square or Pearson tests were used for association or correlation analysis, respectively.

## Results

### Pregnancy-induced malaria recrudescence and poor pregnancy outcome

**Figure 1:**
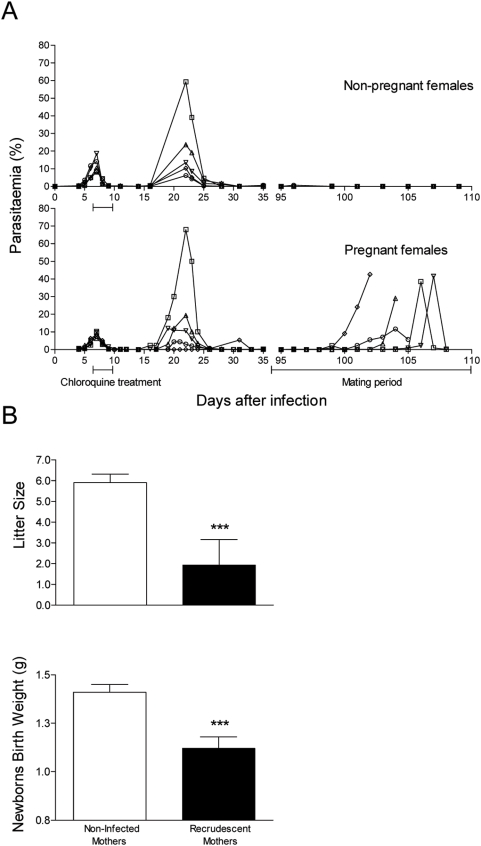
Malaria susceptibility is increased during pregnancy with detrimental effects for the progeny. Representative parasitemia curves of BALB/c females infected with *P. berghei* - GFP (day 0) and treated with chloroquine for 3 days starting at day 7 (Panel A). Parasitemias of females maintained without male (non-pregnants) are represented in the upper plot. The lower plot shows 5 typical parasitemia curves of recrudescent primigravida. In Panel B average litter size and birth weight of 20 litters from non-infected primiparous females are compared with the average litter size of 29 litters and the birth weight average of 6 litters, from recrudescent primiparous females. Error bars represent standard error (***, *p*<0.001).

A total of one hundred female mice were infected with *P. berghei*-parasitized red blood cells and subsequently treated with chloroquine, typically showing a transient parasitemia peak, which eventually resolved. In absence of pregnancy, parasitemia remained essentially subpatent and was not observed thereafter (Figure 1A). In contrast, parasite recrudescence was frequently observed when malaria-treated females become pregnant (Figure 1A), more often after gestation day 14 (G14) but never before G12. We followed the first pregnancy of eighty-four pre-exposed females and found out that forty nine (58%) showed parasite recrudescence induced by pregnancy. Twenty-nine recrudescent females were followed to the end of pregnancy and we observed uncontrolled parasitemia leading to severe malaria and eventually to maternal death in nine of those females (31%) while the remaining controlled the parasitemia peak and were apparently cured. These results confirm the hypothesis that subpatent *P. berghei* infection is exacerbated by pregnancy.

To evaluate the effect of pregnancy-induced malaria recrudescence in the pregnancy outcome we monitored the offspring of primiparous recrudescent females (Figure 1B). The twenty-nine recrudescent mothers had significantly smaller litter sizes (average of 1.9 newborns/litter) as compared to twenty non-infected females (5.9 newborns/litter in average). Likewise, the average birth weight of newborns from recrudescent mothers (1.1 g) was significantly lower when compared to the newborns from non-infected mothers (1.4 g). These findings indicate that recrudescent females show poor pregnancy outcome that is characterized by decreased fetal viability and intra-uterine growth retardation.

### Parasitemia recrudescence correlates with placenta pathology

**Figure 2:**
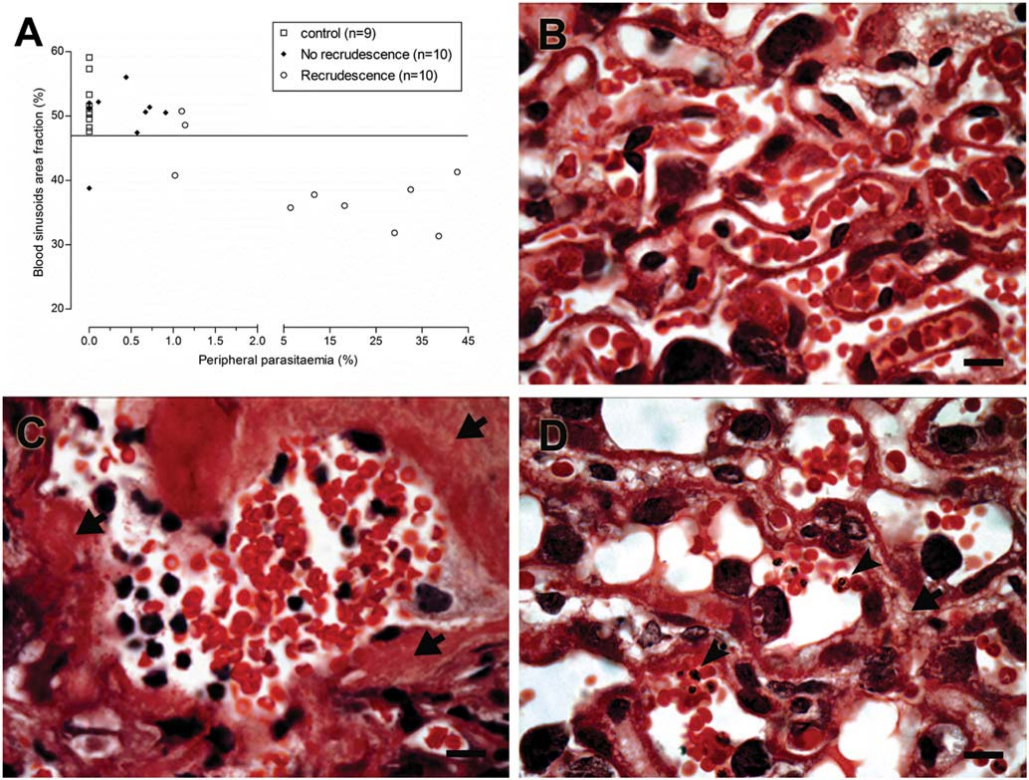
Peripheral parasitemia correlates with the reduction of placental blood sinusoids area. (A) The blood sinusoidal area is plotted against the peripheral parasitemia peak observed in the pregnancy of primiparous females. The area of placental blood sinusoids, expressed as a fraction of the total placental area, was obtained using an automated morphometric procedure as described in Materials and Methods section. In recrudescent females, the degree of parasitemia was correlated with sinusoidal area reduction (correlation coefficient for recrudescent females is 0.45, *P*-value = 0.0012). Representative photomicrograph of placental sections HE stained from non-infected (B) and recrudescent (C–D) mothers. Accumulation of inflammatory cells (C), trophoblast thickening (arrows) and presence of iRBC (D) in blood sinusoids (arrowheads) are evidenced in placenta tissue from recrudescent mothers. Scale bars represent 15 µm in (B–D).

**Figure 3:**
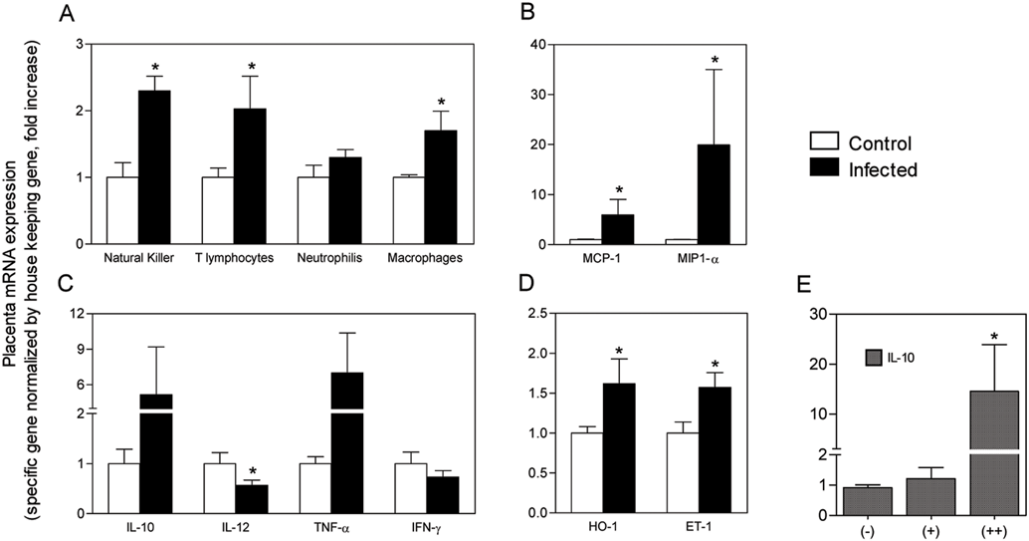
Placenta pathology is associated with altered gene expression of inflammation markers. qRT-PCR of placenta tissue was used to detect the expression of cell type–specific genes indicating infiltration of inflammatory cells: *Klrd1* gene for Natural Killer cells, *Cd3e* gene for T cells, *Ncf2* gene for neutrophils and *Mgl2* for macrophages (A). Placental gene expression was quantified for relevant markers of monocyte/macrophage chemotaxy (B), inflammation mediators (C) and vascular stress (D). RNA expression was quantified in 15 placentas from recrudescent primiparous BALB/c females and in 8 uninfected placentas, collected on G19. In (E) placental *IL10* mRNA expression was separately analyzed in 5 placentas showing moderate pathology (+) and 4 placentas showing severe (++) pathology. Relative quantification was obtained with normalization by β-actin for (A), (C), (D) and (E) and by GAPDH for (B). Results are plotted as fold change over the respective non-infected controls and each bar represents the mean±s.e.m. (*, *P*-value<0.05).

The poor pregnancy outcome in females infected during pregnancy is associated with a placental inflammatory response that leads to marked tissue disorganization [22], and the presence of maternal iRBC of different stages of maturation in the placenta. In recrudescent primiparous females the intensity of peripheral parasitemia was quantitatively correlated with the reduction of the placental vascular spaces (*P*-value = 0.0012) (Figure 2A). In particular, recrudescent females with high parasitemia showed increased reduction of vascular spaces. These results strongly suggest that malaria recrudescence correlated with placental tissue damage (Figure 2C and D) that possibly underlies the observed poor pregnancy outcomes. Furthermore, the expression analysis of cell-type specific genes in placentas from females with recrudescence, revealed increased amounts of inflammatory cells, particularly natural killer (NK) cells, T cells and macrophages (Figure 3A) and up-regulation of macrophages attractant chemokines (MCP-1 and MIP1-α) (Figure 3B).

We also found that the expression of several molecules related to vascular stress, namely hemoxygenase-1 (HO-1) and endothelin-1 (ET-1), was increased in placentas of recrudescent females (Figure 3D). TNF-α expression showed a trend to increase in infected placentas and the balance of the immuno-modulatory molecules IL-12 and IL-10 expression denoted an anti-inflammatory response in the course of the placenta malaria pathogenesis (Figure 3C). In fact, IL-10 expression was particularly increased in placentas where pathology was more intense (Figure 3E).

### Enhanced adhesion of recrudescent *P. berghei* - GFP

**Figure 4:**
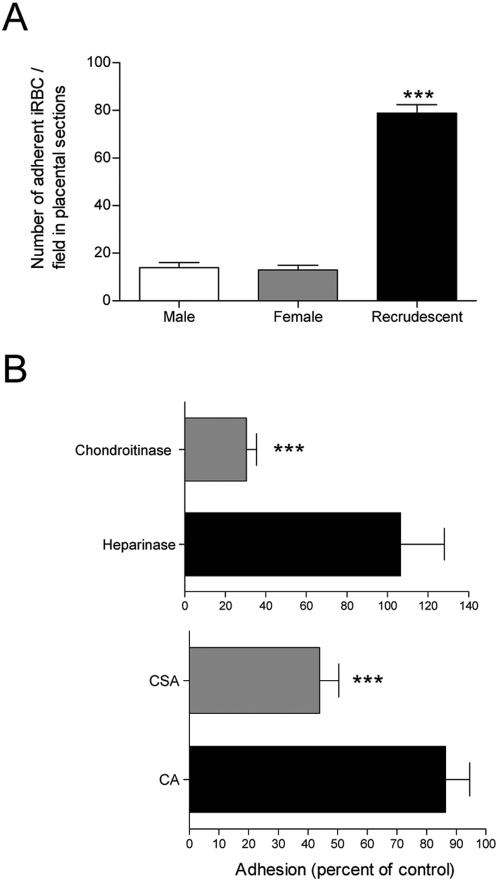
*P. berghei* iRBC from recrudescent females show enhanced adhesion to placenta. iRBC from males, non-pregnant females and recrudescent primiparous females were incubated on uninfected placental sections and the adherent parasitized cells were counted as described in Materials and Methods section (A). Adhesion assays were also performed after pre-treatment of placental sections with chondroitinase ABC or heparinase (negative control) (B, upper plot). Adhesion inhibition assays were carried out by pre-incubating iRBC from recrudescent females with 1 mg/ml concentrations of CSA or CA (negative control) (B, lower plot). In panel B the proportion of bound iRBC is expressed as a percentage of the control (non-treated placentas or non-preincubated iRBC, in upper and lower plots, respectively). Error bars represent the mean±s.e.m. of three independent experiments. (***, *P*-value<0.001).

iRBC sequestration appears to be the pathogenic trigger of the placenta pathology observed in pregnant women and we have previously shown that *P. berghei* iRBC specifically adhere to the mouse placenta tissue. To evaluate the adhesion properties of the recrudescent *P. berghei* we performed adhesion assays on placental sections that compared the adhesion properties of iRBC collected from recrudescent primiparous females with iRBC isolated from infected males and non-pregnant females. Strikingly, the amount of iRBC adhering to the placenta sections was four-fold increased in the samples from recrudescent primiparous females (Figure 4A). The adhesion of the recrudescent parasite was partially inhibited when the placental sections were treated with chondroitinase (70%), and also when the iRBC were pre-incubated with CSA (56%) (Figure 4B). These results indicate that CSA is involved in the adhesion of the recrudescent parasite to the placenta tissue and suggest that the recrudescent *P. berghei* expanding during pregnancy displays enhanced specificity to the placenta and consequently may induce a specific host response to the pregnancy-associated parasite.

### Pregnancy-induced recrudescence and disease severity are reduced in multiparity

**Figure 5:**
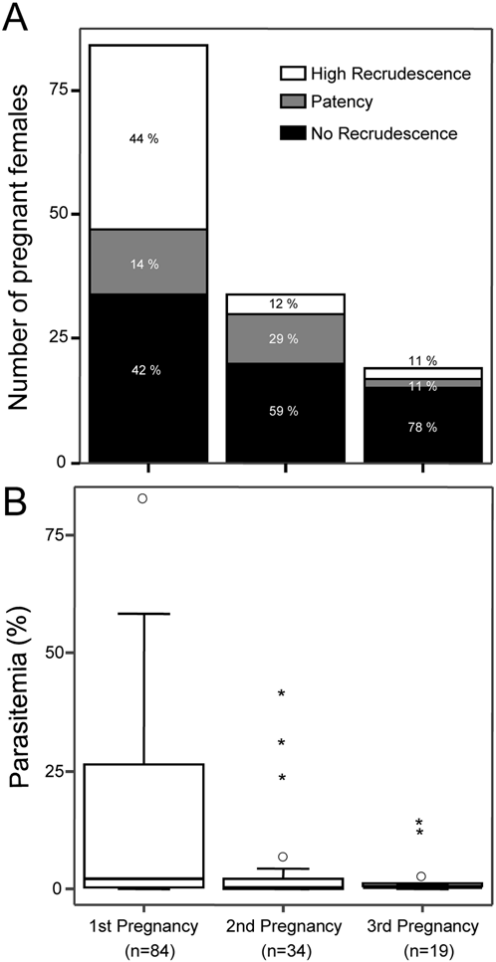
Recrudescence incidence and peripheral parasitemia are decreased in multigravida. (A) Frequency of females with high recrudescence (above 5% parasitemia), patency (parasitemia between 1% and 5%) and no recrudescence (parasitemia <1%) according to parity. Recrudescence incidence is significantly associated with parity (*P*-value = 0.001, Chi-square test). (B) Box-plots illustrate the range of the peripheral parasitemia peak according to parity. The parasitemia peak in the first pregnancy was significantly different from the second (*P*-value = 0.004) and third pregnancies (*P*-value = 0.006). * and ^○^ represent extremes and outliers, respectively.

**Figure 6:**
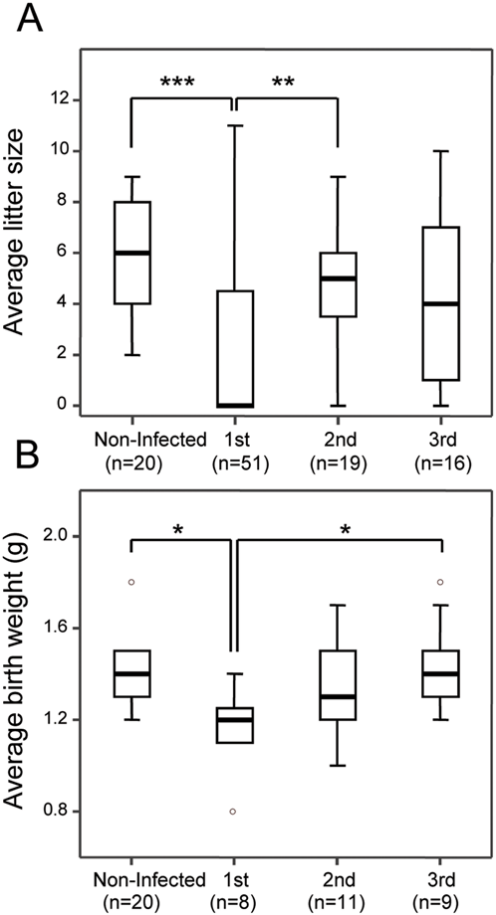
Reduced adverse pregnancy outcomes in multigravida. Box-plots of the average litter size (A) and average newborn birth weight (B) according to parity (first, second and third pregnancy). Pregnancy outcome was significantly different in primigravida as compared to multigravida and non-infected pregnant females (***, *P*-value<0.001; **, *P*-value<0.01; *, *P*-value<0.05).

To test whether pregnant females were able to mount a response upon exposure to the recrudescent parasite, we followed up the fate of thirty-two primigravida in subsequent pregnancies. The pregnancy-induced peripheral parasitemia peak was graded as high recrudescence, if higher than 5%, and as patency, if between 1% and 5%, while the pregnant females with less than 1% of iRBC as detected by FACS analysis were declared non-recrudescent. We found that the aggregate incidence of high recrudescence and patency significantly decays from the first (59%) to the second (41%) and third pregnancy (22%) (Figure 5A). The reduced incidence of cases with high pregnancy parasitemia peaks (more than 5% of iRBC) was particularly striking and close to a four-fold reduction from the first (44%) to the second pregnancy (12%). Accordingly, the level of parasitemia also decreased significantly when comparing first (14.5%±19.2%), second (3.9%±9.4%) and third pregnancy (2.0%±4.1%) (Figure 5B). Also, the litter size and the newborn birth weight were lower in the first pregnancy but recovered and approximated normal levels in subsequent pregnancies (Figure 6). Furthermore, analysis focused on recrudescent females revealed that maternal mortality associated to recrudescence decreases with parity (Table 1) suggesting a decrease in disease severity. These results suggest that females that are repeatedly exposed to recrudescent parasites during pregnancy develop a protective response that tends to control parasite recrudescence and placental malaria during subsequent pregnancies.

**Table 1:** Disease severity and pregnancy outcome in *P. berghei* recrudescent females according to parity.

Parity	Pregnant females	Average parasitemia peak (%)	Maternal mortality (%)	Litter size^a^	Newborns Birth weight (g)^a^
Primigravida	29	22.7	31	1.9±3.0 (29)	1.1±0.2 (6)
Second Pregnancy	9	10.7	20	5.6±2.1 (9)	1.3±0.2 (7)
Third Pregnancy	3	3.4	0	6.7±1.2 (3)	1.3±0.1 (3)
Non-Infected	20	___	0	5.9±2.2 (20)	1.4±0.2 (20)

a Mean±stdev (number of litters analyzed).

PAM protection in multigravida is not attributable to the age of the pregnant females as we observed that pregnancy-induced recrudescence incidence in primigravida was not reduced at older ages. In particular, females infected under 20 weeks of age presented about 55% of recrudescence and in the group of older females, with more than 20 weeks, the recrudescence was about 65%. Furthermore, pregnancy-induced recrudescence seems to be uncorrelated with the period between infection and the first pregnancy, since we were able to observe recrudescence 40 weeks after infection. These data suggest that both the age of the mother and the duration of subpatent parasitemia are not determining factors in triggering parasite recrudescence.

**Figure 7:**
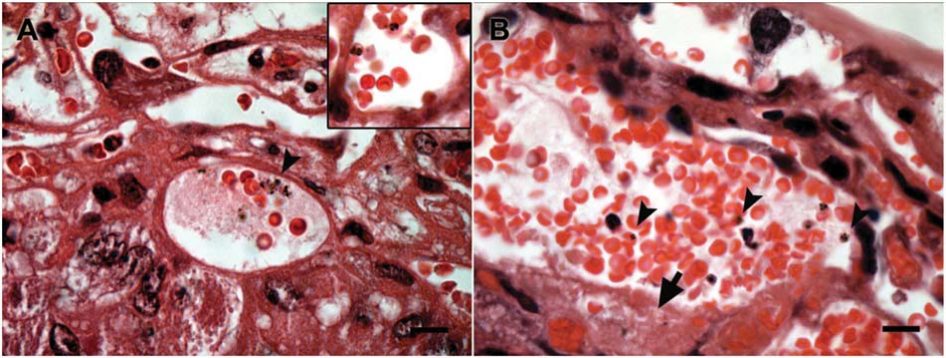
Occasional placenta pathology in non-recrudescent pregnant females. Photomicrographs of HE-stained placental sections of sporadic cases of placental pathology in non-recrudescent females. The figure shows presence of iRBC adhered to the syncytiotrophoblast layer (A, insert) and in blood sinusoids (arrowheads) as well as trophoblast thickening (arrow). Scale bar represents 15 µm.

It is worth noting that, regardless the number of previous pregnancies, the placentas from recrudescent females typically showed iRBC in the maternal blood spaces, inflammatory infiltrates, erythroblast accumulation in the fetal blood, placenta architecture disruption and trophoblast basal membrane thickening (data not shown). We noted that among non-recrudescent females the litter size was below normal levels and that irrespective of parity, about ten percent died during pregnancy or shortly after delivery (Table 2). The analysis of non-recrudescent placentas in some cases revealed the presence of iRBC and tissue lesions resembling the pathology observed in recrudescent females (Figure 7). These data suggest that albeit at low frequency, placental malaria occurs in absence of peripheral parasitemia recrudescence. Together, this study suggest that pregnancy-associated malaria evoked by recrudescent *P. berghei* is attributable to parasites with enhanced specificity for the placental tissue that are able to induce an inflammatory response during pregnancy and a cumulative protective response in multigravida that had experienced recrudescences induced by pregnancy

**Table 2:** Disease severity and pregnancy outcome in non-recrudescent females according to parity.

Parity	Pregnant females	Maternal mortality (%)	Litter size^a^	Newborns Birth weight (g)^a^
Primigravida	22	12	3.2±3.3 (22)	1.4±0.2 (2)
Second Pregnancy	10	11	3.8±2.4 (10)	1.4±0.3 (4)
Third Pregnancy	13	8	3.5±0.2 (13)	1.5±0.7 (6)
Non-Infected	20	0	5.9±2.2 (20)	1.4±0.2 (20)

a Mean±stdev (number of litters analyzed).

## Discussion

In this paper we analyzed a mouse model of pregnancy-induced malaria recrudescence in which the intensity of parasite recrudescence showed to be quantitatively correlated with the placenta pathology and the recrudescence incidence decreased with parity. Furthermore, we showed that the recrudescent parasite displays enhanced adhesion to the placenta suggesting that *P. berghei* could be helpful in investigating and clarifying the intricate mechanisms governing the increased risk of malaria recrudescence induced by pregnancy [34], [35].

Our experiments in mice clearly support the possibility that PAM in pre-exposed individuals does not require re-infection and suggest that malaria recrudescence during pregnancy can be caused by pregnancy-specific mechanisms that remain to be identified. These results are well in line with early findings by van Zon and Eling [20], [32] and more recently by Megnekou et al. [23] reporting *P. berghei* peripheral recrudescence in pregnant female mice that acquired the infection before conception. Moreover, in our analysis parasite recrudescence was never detected before gestation day 12 (G12), and most frequently parasitemia arose after G14. Placental development studies show that at G10.5 maternal blood starts to be evident in the labyrinth but only by G12.5 the definitive placenta becomes functional [38]. These observations converge to the notion that the placenta plays a critical role in development of murine PAM, possibly by promoting parasite recrudescence.

One of the currently proposed roles for the placenta in PAM pathogenesis is to provide new ligands that are recognized by the iRBC. We have previously shown that *P. berghei* binds CSA and hyaluronic acid [22] and in this study we demonstrated that iRBC collected from recrudescent females displayed a marked enhancement of CSA binding properties. The role of hyaluronic acid in the iRBC-placenta interaction has been to a certain extent controversial [33]–[35], but we observed that hyaluronidase and pre-incubation with hyaluronic acid inhibit the binding of recrudescence iRBCs to the placenta (data not shown). The adhesion assays clearly indicate that recrudescent *P. berghei* iRBC have higher ability to adhere to the placenta, raising the hypothesis that *P. berghei* expanding during PAM is positively selected by the ability to bind placental ligands. This hypothesis is supported by very recent work showing that pregnant mice acquire immunity specific to the recrudescent parasite [23]. This would parallel previous reasoning suggesting that in human PAM specific *P. falciparum* variants, such as the variant expressing the PfEMP1 molecule encoded by the *var* gene *var2csa*, are expanded via the increased cytoadherence of the iRBC to the placental receptors, prominently the CSA [39]. It is noteworthy that *P. berghei* variant antigens were not described so far, even though antigenic variation had been shown in other murine malaria species, such as *P. chabaudi* AS [36], [37]. Nevertheless, our data opens the possibility that murine PAM entails an overrepresentation of *P. berghei* - iRBC displaying parasite components that mediate the iRBC-placenta interactions. Thus, this model provides an opportunity to study the molecular mechanisms that promote the expansion of placenta-binding parasites also observed in human PAM.

In the course of *P. falciparum* infections, the placenta can harbor a striking density of parasites, macrophages, haemozoin and excess of fibrinoid deposits associated to morphologic alterations, such as syncytiotrophoblast necrosis and trophoblast basement membrane thickening [38] that would be harmful to the developing fetus, as the placental exchanges of respiratory gases and nutrients became difficult and reduced. We previously demonstrated that these characteristics can also be observed in the model system using BALB/c mice and *P. berghei* ANKA [22]. A remarkable pathological finding in *P. berghei* ANKA recrudescent placentas was the reduction of blood sinusoids space that is attributable to placental tissue thickening (Figure 2D). In addition, we showed that the reduction of the blood sinusoidal space is highly dependent on the parasitemia level (Figure 2A), reinforcing the notion that the parasite has a pivotal role in the genesis of the placental pathology.

Cytoadherence of *P. berghei*–infected erythrocytes to receptors expressed on the syncytiotrophoblast surface is considered to contribute to the described placental disorders, but it is not a sufficient condition for pathogenesis [39]. Placental malaria studies propose that the observed intervillositis is mostly an immunopathologic process, due to cytokines and chemokines production and leading to the activation of the syncytiotrophoblast [40], [41].

Monocytic/macrophagic infiltrate has been considered a hallmark of *Plasmodium*-infected placentas [39], [42], [43]. We had previously verified that infected *P. berghei*-GFP mouse placentas had an accumulation of mononuclear cells [22]. It is important to remark that in mouse placentas we observed low degree of massive chronic intervillositis as compared to reported observations in infected human placentas [44]. Possibly this difference is related to the short pregnancy time span that may condition the inflammatory process in the mouse as compared to human pregnancy. The expression profile of inflammatory genes suggests that, at the end of the pregnancy period, the placentas of recrudescent mice undergo a strong inflammatory process. This is inferred from the significant increase of cell-type specific markers for NK cells, T lymphocytes and macrophages as well as increased expression of chemoattractant factors (Figure 3A and B). Our results are in accordance with a recent study in Kenya showing NK and T cell infiltration in *P. falciparum*-infected human placentas [45]. It remains to be resolved whether T cell infiltration corresponds to activated effector T cells or to T regulatory cells as part of a placenta anti-inflammatory response. Interestingly, in our model, IL-12 expression in the placenta was significantly decreased (Figure 3C), while the expression of TNF alpha and IL-10 was increased. This down-regulation of IL-12 production, presumably from its main cell sources was suggested to be due to the inhibitory effects of hemozoin [46]. These observations allow the speculation that the strong local inflammatory environment generated by the iRBC adhesion is counteracted by a systemic anti-inflammatory response. In fact, we noted that the up-regulation of *IL10* expression was correlated with the severity of placenta pathology (Figure 3E). Coincidently, IL-10 levels in the serum have been associated with poor pregnancy outcomes and was suggested as a biomarker for placenta inflammation in pregnant women [47]. Placenta microcirculation is in part controlled through a fine balance between different vasoconstrictors such as ET-1, and vasodilators, namely nitric oxide or HO-1 enzyme [48], [49]. Recrudescent placentas showed increased ET-1 and HO-1 mRNA expression, suggesting that the placenta vasculature is exposed to abnormal vasoactive regulation. Interestingly, we found that in some non-recrudescent females, the placentas could harbor a very low density of iRBC, which nevertheless seemed high enough to trigger placenta pathology (Figure 7). We speculate that these pathologic mechanisms would explain the death of pregnant females that do not show peripheral parasitemia but exhibit placental pathology.

There is solid epidemiological evidence from high-endemicity malaria regions that the incidence of PAM in women is parity-dependent [1]. In mice, reduced recrudescence has been observed in multigravida after anti-malaria treatments during the first pregnancy [36]. In the PAM mouse model described here we have also found that the disease incidence and severity decreased with parity. Nevertheless, we did not find a correlation between the intensity of the parasitemia peak in the pre-mating period and the occurrence of pregnancy-induced recrudescence in the first pregnancy, implying that such exposure did not confer PAM protection. These observations compelled us to hypothesize that PAM protection is acquired through repeated exposure to the recrudescent parasite on line with a recent report on accumulation of immunity to PAM over several pregnancies and recrudescences [24]. In this context, a possible extension of this model could be the repeated pre-exposure of females before pregnancy. This could help to evaluate the robustness of protection conferred by non-pregnant parasites.

Here, we report on the analysis of a mouse model of pregnancy-induced parasite recrudescence that displayed features resembling those of PAM in women living in high-endemicity regions. This work further supports the notion that this mouse model may become a helpful tool to dissect the molecular and cellular components involved in parasite recrudescence during pregnancy and to identify the details of the immunological mechanisms conferring PAM protection.
